# Attitudes, Beliefs, and Behaviors about Cigarette-Butt Littering among College-Aged Adults in the United States

**DOI:** 10.3390/ijerph19138085

**Published:** 2022-07-01

**Authors:** Thomas Webler, Karin Jakubowski

**Affiliations:** 1 Department of Environmental Studies, Sustainability, and Geography, Keene State College, Keene, NH 03435, USA; 2 Department of Biology and Environmental Science, University of New Haven, West Haven, CT 06516, USA; kjakubowski@newhaven.edu

**Keywords:** marine debris, cigarette butts, littering behavior, survey, attitudes

## Abstract

This study reports attitudes, beliefs, and littering behaviors of 7532 college-aged cigarette smokers from across the United States. Four behavioral variables were measured: littering of last cigarette butt, number of butts littered in past 24 h, littering in past month, and ever having littered. Questions about beliefs centered on whether cigarette butts are biodegradable, if butts were harmful to the environment, and if butts are considered to be litter. One attitudinal question focused on whether seeing butts on the ground was bothersome. Littering was most likely among people who believed butts were biodegradable, believed they are not harmful to the environment, do not believe butts are litter, and among those with the attitude that littered butts are not bothersome. Logistic regression analyses found that the strongest influence on littering behavior was the attitude that seeing butts was bothersome. The second-strongest driver was the belief that butts are litter.

## 1. Introduction

According to the latest littering study by Keep America Beautiful, cigarette butts remain the most commonly littered item, comprising nearly 20% of all litter. The 2021 report estimates that 9.7 billion cigarette butts are littered in the United States each year, and four billion of these are in waterways [1]. Many butts littered on land are likely mobilized to oceans [2]. Cigarette butts are made of cellulose acetate, a plastic. Hence, they contribute to the volume of plastics entering the world’s ocean each year [3,4].

According to one estimate quoted by the Centers for Disease Control and Prevention (CDC), about 8% of 18–24-year-old adults in the United States are cigarette smokers [5]. College-aged adults are an important population because their behaviors will likely have many years of influence. As the next generation of parents, their behaviors are also likely to be passed onto the following generation [6].

This study investigated the attitudes, beliefs, and behaviors of college-aged cigarette smokers in the United States. It sought to better understand the factors associated with littering cigarette butts. Using a panel from the research survey company, Qualtrics, we collected data from 7532 college-aged cigarette smokers in the United States about their beliefs, attitudes, and behaviors related to littering cigarette butts.

The structure of this paper is as follows. It begins with a review of the literature on the importance of plastic and cigarette-butt litter, in particular on marine ecosystems, and a review of the literature on smokers’ attitudes, beliefs, and littering behaviors. The methods section explains the survey design and recruitment strategy. Multivariate logistic regressions that identify beliefs and attitudes that were predictive of behavior are reported. The discussion section interprets the findings in the context of previously published research and elaborates on their relevance for changing littering behavior.

## 2. Background

### 2.1. Cigarette Butts as Marine Debris

In 2016, over 5.5 trillion cigarettes were sold worldwide [7,8]. It is estimated that >80%, or over 4 trillion cigarette butts are littered each year. Litter surveys consistently find cigarette butts to be the most common form of litter worldwide. In the United States, some 263 billion cigarettes were sold (ibid), and, assuming 80% were disposed of improperly, this would mean about 77 million pounds of cigarette-butt litter are dropped on the ground each year. As public health efforts to reduce exposure to secondhand smoke indoors push smokers out of doors, the percentage of cigarette butts disposed of on the ground is likely increasing. This is certainly the case at many college campuses and professional offices, where piles of butts can be found on the ground in areas just outside the doorway, 25 feet from a building as required in many instances, or just beyond the property line of the institution (in cases where the institution has banned smoking). Many of these butts find their way into storm sewers and rivers, which transport debris to the oceans [2]. Ocean Conservancy found that cigarette butts were the most common form of litter collected in their international coastal cleanup, with 1.8 million butts collected in 2017 [9]. While a formidable number, this amounts to less than one-millionth of all cigarette butts littered each year.

In the ocean, the cellulose acetate (that is, the plastic making up a cigarette filter) joins the massive collection of other anthropogenic litter already floating on or suspended in the water. Plastics are the main component of this litter. The volume increases yearly [10] and is projected to increase by an order of magnitude within the next decade [3]. Moreover, used cigarette filters transport to marine environments numerous hazardous chemicals from the tobacco smoke that is trapped in the filters [11].

The biological and ecological implications of used cigarette filters and other plastics are profound [12]. 580 species of invertebrates, fish, birds, turtles, and mammals have been found to suffer deleterious effects from plastics. Large pieces can lead to entanglement or mechanical blockage of the alimentary canal, leading to starvation. Ingested microplastics—including those that originate from cigarette filters—can produce toxic effects, and nanoplastics have been found to transit across cell walls [13]. Micro- and nanoplastics pose particular risks to zooplankton, filter feeders, and invertebrates [14]. They can also be drawn into the gill cavities of fish and crustaceans [15]. Ingestion of plastics is most noticed in pursuit-diving seabirds and surface-seizing seabirds, although it is also found in turtles, fish, crustaceans, snails, and mammals [16,17,18]. In general, direct ingestion of plastics is believed to produce sublethal effects rather than direct death [19]. A common sublethal effect is lack of nutrition due to the stomach being partially occupied with undigested plastics or the intestinal wall being coated with plastic film. Marine plastic debris is also associated with complex mixtures of chemicals, some added during manufacturing and others collected through use (such as cigarette filters) or adsorbed from other contaminants in the environment post-use. Floating microplastics can accumulate and concentrate toxic chemicals such as polychlorinated biphenyls (PCBs) that are diluted in the marine environment [20]. These cocktails of hazardous chemicals are transported inside the body through ingestion and there they produce numerous physiological effects including illness, mutagenicity, and death [20,21]. This is a threat to hundreds of species of marine animal life. In addition, the presence of used cigarette butts contaminates habitat. Moriwaki et al. found evidence that of arsenic, nicotine, polycyclic aromatic hydrocarbons, and heavy metals in soils where cigarette butts were discarded [22]. It is likely that marine habitats are similarly impacted. One cigarette butt was found to be capable of contaminating a thousand liters of water at concentrations above no effect levels [23].

### 2.2. Smokers’ Beliefs and Littering Behaviors

The behavioral literature on littering behaviors is extensive. Littering is known to depend on many things, including the type of object littered; whether the ground is already littered; availability of trash receptacles; type of location; and time of day, to name a few (for reviews, see [24,25]). However, when it comes to littering of cigarette butts, the literature is much more limited.

The largest observational study of smokers’ littering behavior in public spaces found that 57% of people who were observed smoking littered one or more butts, and overall, 65% of cigarette butts were littered [26]. These are low estimates because 27% of smokers observed had not finished smoking when they left the observation area. This study also reported on the age of smokers (but only included individuals over 21 years old), gender, time of day, and whether the person was alone or in a group. The only significant correlation was with age—younger people littered butts more frequently. Another observational study of public spaces found that smokers in Victoria University in Wellington NZ littered 98.7% of butts [27]. A study of 219 smokers in downtown Wellington, NZ found that 76% of smokers littered their butts [28]. Neither of these studies in New Zealand collected information about the litterers. 

Rath et al. conducted one of the few non-tobacco-industry studies that measured cigarette smokers’ beliefs toward cigarette-butt litter and their cigarette-butt-disposal behavior [29]. This was a self-report study, not an observational study, which has strengths and weaknesses. One of the strengths is that it can measure littering behavior in private as well as public spaces. Their survey study of 2000 smokers in the United States found that 55.7% reported littering butts in the past month and 74.1% reported littering butts at least once in their lifetime. A recent attempt to influence smokers’ littering behavior used anti-littering messaging on cigarette packs [30]. For five weeks researchers added labels to 719 smokers’ cigarette packs. The anti-littering labels included statements of fact about cigarette butts and litter (e.g., “Cigarette butts don’t biodegrade. Please do not litter”). A second experimental group received stickers with statements about the chemical composition of cigarette smoke or health effects (e.g., “Cigarette smoke contains arsenic. This causes heart damage”). There were no baseline data collected, nor was there a control group. Anti-littering messages were associated with greater intentions to not litter but not less littering.

### 2.3. Beliefs about Biodegradability and Environmental Harmfulness

Cellulose acetate, the plastic comprising cigarette filters, is not biodegradable under normal natural conditions [31]. However, in an ocean environment where it is exposed to sunlight and wave action, the cellulose acetate in cigarette butts does disintegrate into smaller and smaller pieces. Due to its chemistry, cellulose acetate can also collect other types of chemicals in the ocean, including carcinogens such as PCBs. Small or even microscopic pieces of cigarette filters, contaminated with toxins from the tobacco or the environment, become available to different organisms in the food web. When they are consumed, the animal also ingests any accompanying toxins [32,33].

Rath et al. included measures of knowledge (which is the most common item collected in beach clean-ups?) and beliefs (cigarette butts are toxic, biodegradable, harmless for people to eat, harmless for marine organisms to eat, dangerous to throw in a trash can, cigarette butts are litter). They investigated which of these most strongly related to littering behavior. The strongest relationship was found to be with the belief that butts are litter. Smokers who thought of cigarette butts as litter were 3.68 (95% CI 2.04 to 6.66) times more likely to properly dispose of their butts.

Morgan et al. asked participants if they knew that cigarette butts were the most common kind of litter and if they knew that cigarette butts do not biodegrade, but they did not report on the effectiveness of these variables on intention to litter or littering behavior [30]. After five weeks of the trial, 68% of people who received the anti-littering messages knew that butts were not biodegradable compared to 48% in the chemical message group.

## 3. Methods

The research design was reviewed and approved by human-subject review boards at Keene State College (New Hampshire) and University of New Haven (Connecticut). The informed consent was acquired before participants completed the survey.

### 3.1. Survey Design

Five littering-behavior questions were asked. The first two were combined in the analysis into a single measure of “ever littered”. The four measures served as the dependent variables in the analysis.

- Did you ever toss a cigarette butt on the ground? (yes/no)
- Did you ever toss a cigarette butt out a car window? (yes/no)
- In the past month have you ever disposed of a cigarette butt in any of these places (select all that apply)? (On the ground, out of a car window, in a trash can, in a public ashtray, pocketed it, in a sewer drain, other)
- How did you dispose of the last cigarette butt you smoked? (On the ground, out of a car window, in a trash can, in a public ashtray, pocketed it, in a sewer drain, other)
- In the past 24 h how many cigarette butts have you dropped on the ground, tossed out of a car window, or thrown into a sewer drain? (slider from zero to ten or more)

Attitudes are known to precede intention to act in many models of environmental behavior, but they have not been measured in previous studies of littering by smokers. Attitudes toward cigarette-butt litter were measured by asking respondents to agree or disagree with the following statement: “Seeing cigarette butts on the ground bothers me”. Three questions measured beliefs about butts and littering: “cigarette butts are biodegradable”, “cigarette butts are litter”, and “cigarette butts are harmful to the environment”. In addition, measures of smoking intensity, age, race, gender, and ethnicity were made. One question asked about carrying a personal pocket ashtray (every day, most days, occasionally, never). Halfway through the study we added one agree/disagree question about social norms: “There is nothing wrong with throwing a cigarette butt on the ground”.

### 3.2. Recruitment

Qualtrics was hired to recruit respondents for the study. Qualtrics is a research survey company that has access to multiple panels of research subjects. They were instructed to filter for age group (18–24 year olds), cigarette smoking, and US residency.

### 3.3. Analysis

Chi-squared tests were used to estimate relationships among nominal variables. Logistic regression and multiple regression were used to estimate relationships between dependent variables and our four independent variables. Only variables that were statistically significant in the logistic regression were entered into the multiple regression analysis. Odds ratios and 95% confidence intervals were calculated.

## 4. Results

### 4.1. Demographics

After completing the informed consent page, 7532 individuals in our targeted age group (18–24-year-old smokers) competed the survey. Slightly fewer males (49.4%) than females (49.7%) participated, but the percentages were very close to US Census estimates of 51.2% male and 48.8% female for this age group. Less than 1% reported a gender other than male or female or refused to answer that question.

Of the respondents, 20.4% reported they were of Hispanic origin, which compares to a national average of 16.8% for all people over 18 years old. Table 1 shows the racial make-up of our sample and compares it with Census results for all people over 18 years.

### 4.2. Smoking and Littering Behavior

Over 40% reported smoking 1–5 cigarettes a day, and 4% said they smoked 20 or more cigarettes a day (see Table 2). We estimated that all together, our respondents smoked 55,000 cigarettes a day or 20 million a year.

*Personal pocket ashtray.* Smokers sometimes carry a personal pocket ashtray. We found that 49% of men and 33% of women reported they carry one “always” or “most days”. About 25% of men and 25% of women reported carrying one “occasionally”. Over 40% of women and 25% of men reported never carrying one. Race was not independent (*X*^2^ (15, *n* = 7358) = 71.5, *p* < 0.001). Black/African-American participants (46%) were more likely than White participants (40%) to carry a personal ashtray (*X*^2^ (3, *n* = 6461) = 32.5, *p* < 0.001).

*Past littering behavior*. A large majority (72%) reported tossing a butt to the ground at least once in their lifetime (70% females, 77% males) while 64% reported tossing a butt out a car window at least once in their lifetime (63% females, 66% males). Just over half the respondents reported tossing a butt to the ground at least once in the past month and slightly under half reported tossing one from a car window. Less than one in five reported tossing a butt in a sewer drain in the past month. Interestingly, only 60% recalled disposing of a butt properly in a trash can or a public ash tray in the past month. Pocketing butts was less popular.

Carrying a pocket ashtray was associated with lower littering behaviors. Those who never carry a pocket ashtray are almost five times more likely to have tossed a butt to the ground and 2.7 times more likely to have tossed one out a car window in the past month.

**Figure 1:**
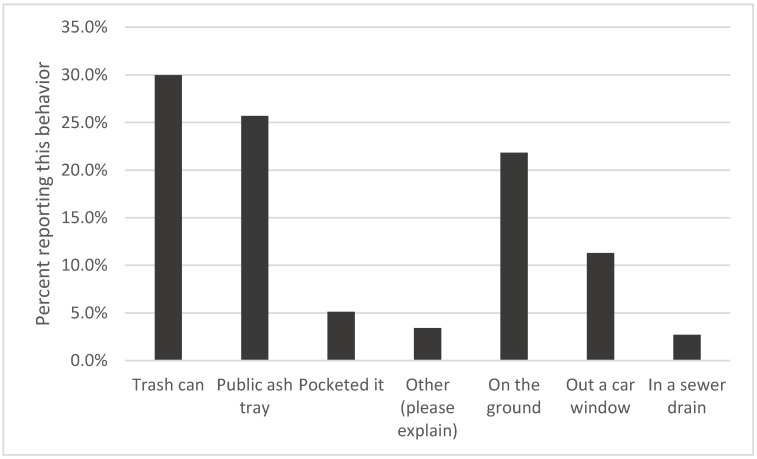
Last cigarette-butt-disposal practices (*n* = 7532).

We asked respondents to report how they disposed of the most recent cigarette butt that they smoked outdoors (see Figure 1). We distinguished between proper disposal (trash can, public ash tray, or pocketed it) and improper disposal (on the ground, out a car window, or in a sewer drain). Less than ten percent reported “other” disposal choices. A review of the “other” answers shows that they are mainly personal ashtrays, buckets, coffee cans, water bottles, and so on. We classified these as proper disposal methods. In summary, 64% of people reported disposing of their most recent butt properly, while 36% reported improper disposal. Again, people who always, or most days, carried a pocket ashtray littered less than others (*X*^2^ (15, *n* = 7276) = 157, *p* < 0.001).

**Figure 2:**
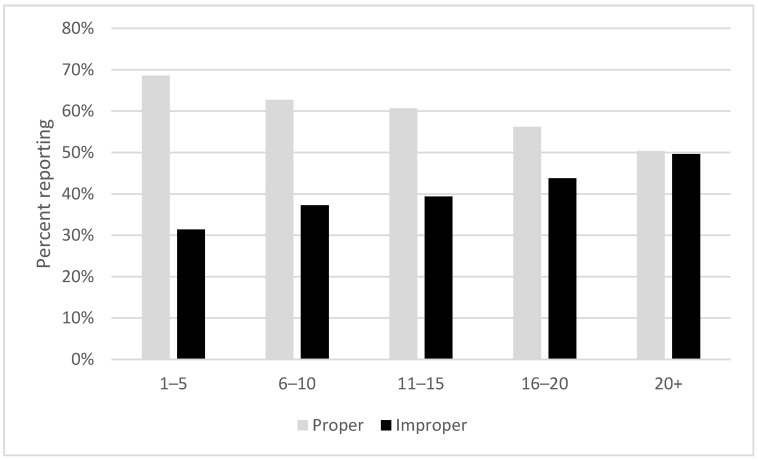
Disposal of last cigarette smoked by daily consumption, normalized to size of each group (*n* = 7532).

We found that people who smoked fewer cigarettes a day were more likely to have disposed of their last butt properly (see Figure 2) (*X*^2^ (60, *n* = 7532) = 927.5, *p* < 0.001) and the effect waned for heavier smokers. Those who smoked more than a pack a day were equally as likely to have disposed of their last butt properly as improperly.

**Figure 3:**
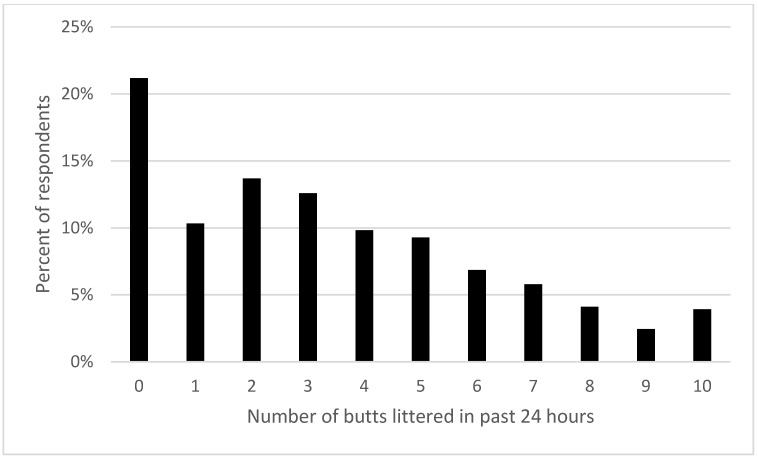
Percent of respondents reporting number of butts improperly disposed in past 24 h (*n* = 7532).

We asked people to report, for all the cigarettes they smoked in the previous 24 h, how many butts they tossed on the ground, threw out a car window, or dropped in a sewer (i.e., disposed improperly or littered, see Figure 3).

**Figure 4:**
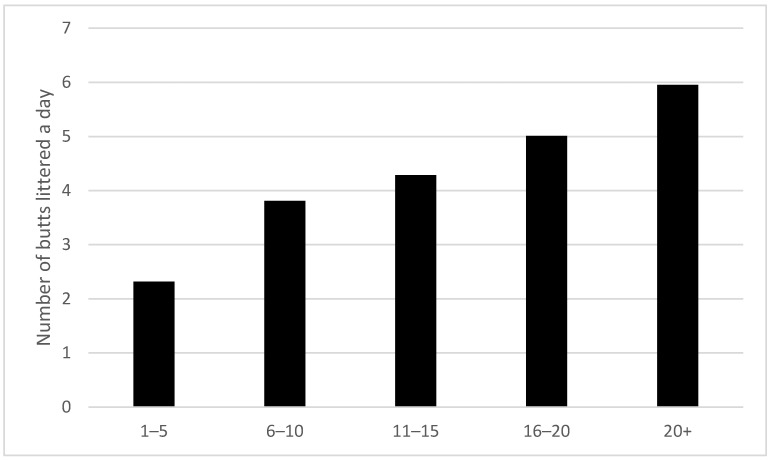
Average number of butts littered per person in past 24 h by number of cigarettes smoked a day (*n* = 7532).

Our 7532 respondents reported improperly disposing of 25,380 butts in the previous 24 h, or about 46% of the daily number of cigarettes smoked. (Note that if this 24-h period was repeated daily, our 7532 respondents would litter over 9.2 million cigarette butts each year or 1230 butts a year per person). People who smoked more also reported littering more butts (*X*^2^ (40, *n* = 7532) = 1433, *p* < 0.001). The rate of littering increased from 2.3 to 6 butts per day as daily consumption of cigarettes increased (see Figure 4).

**Figure 5:**
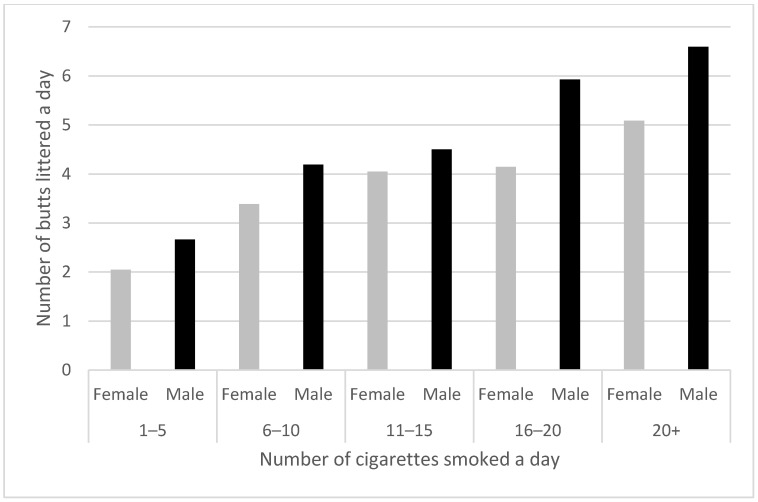
Average number of cigarette butts improperly disposed of by women and men according to number of cigarettes smoked each day (*n* = 7453).

Women littered fewer butts than did men (*X*^2^ (10, *n* = 7463) = 277, *p* < 0.001). The effect was found in all the smoking-rate groups (see Figure 5). 

**Figure 6:**
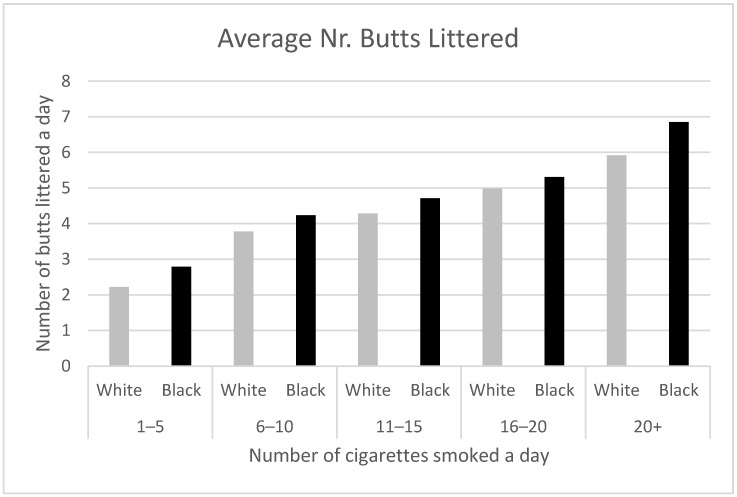
Average per person number of cigarette butts improperly disposed of by race according to number of cigarettes smoked each day (*n* = 6461).

There was no difference in littering rates with ethnicity (*X*^2^ (10, *n* = 7292) = 15.7, *p* = 0.11), but compared to White participants, Black/African-American participants had higher average rates of littering cigarette butts in the past 24 h. This held true across all smoking-intensity groups (see Figure 6).

### 4.3. Attitudes, Beliefs, and Behaviors

*Beliefs and attitudes toward cigarette butt litter.* When asked to respond to the statement, “Cigarette butts are litter”, 79% of respondents agreed while 9% disagreed and 11% had no opinion. Men were slightly more likely than women to agree that cigarette butts are litter (*X*^2^ (1, *n* = 6886) = 9.6, *p* = 0.002). There was no relationship with ethnicity (*X*^2^ (1, *n* = 7532) = 0.8, *p* = 0.36), but White participants were more likely to agree that cigarette butts are litter than Black/African-American participants (*X*^2^ (1, *n* = 7532) = 11.7, *p* < 0.001). People who smoked less were more likely to agree that butts are litter than people who smoked more (*X*^2^ (4, *n* = 7532) = 28.7, *p* < 0.001).

**Figure 7:**
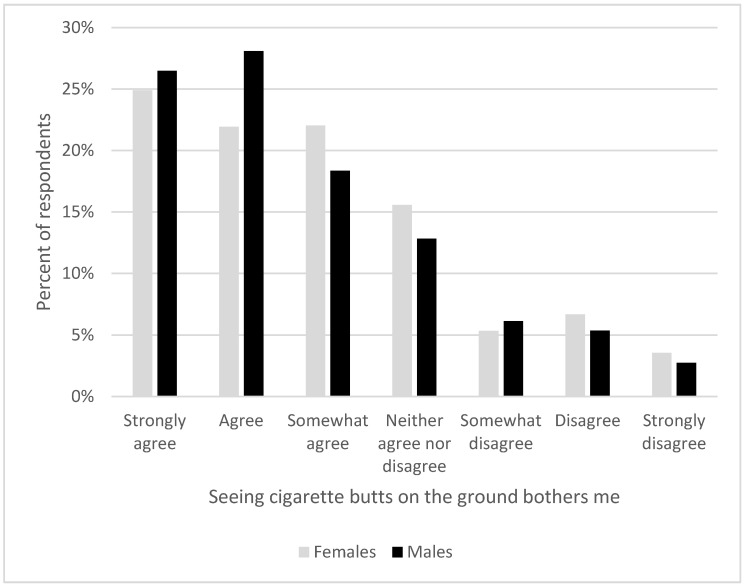
Responses, by gender, to the statement, “Seeing cigarette butts on the ground bothers me”. (*n* = 7532).

When asked to respond to the statement, “Seeing cigarette butts on the ground bothers me”, most (71%) agreed while only 15% disagreed. Gender differences were significant (*X*^2^ (6, *n* = 7532) = 63.9, *p* < 0.001) and mixed. Men were more likely to strongly agree, agree, and somewhat disagree, while women were more likely to somewhat agree, neither agree or disagree, disagree, and strongly disagree (see Figure 7). Race and ethnicity were related to this attitude (*X*^2^ (6, *n* = 6460) = 32.6, *p* = < 0.001) and White participants were significantly more likely to agree that seeing cigarette butts on the ground bothered them than were Black/African-American participants (*X*^2^ (1, *n* = 5577) = 28.2, *p* < 0.001). Hispanic participants were more likely to agree than were non-Hispanic participants (*X*^2^ (6, *n* = 7292) = 23.5, *p* = 0.0014). Participants who smoked less were more likely to report being bothered by cigarette-butt litter than those who smoked more (*X*^2^ (24, *n* = 7532) = 143, *p* < 0.001). Participants who carried a personal pocket ashtray were also more likely to report being bothered by seeing cigarette butts on the ground (*X*^2^ (18, *n* = 7532) = 1011, *p* < 0.001).

When asked to respond to the statement, “There is nothing wrong with throwing butts on the ground”, 35.4% of respondents (*n* = 4216) agreed while 50.6% disagreed. Men were more likely than women to agree (*X*^2^ (6, *n* = 4189) = 87.4, *p* < 0.001). Non-Hispanic participants were more likely to agree than Hispanic participants (*X*^2^ (6, *n* = 4219) = 20.8, *p* = 0.002). Black/African-American participants were more likely than White participants to agree (*X*^2^ (6, *n* = 3783) = 48.7, *p* < 0.001). The more people smoked, the more likely they were to agree that there is nothing wrong with littering butts (*X*^2^ (24, *n* = 7532) = 184, *p* < 0.001). Based on this analysis, the person most likely to believe that there is nothing wrong with throwing butts on the ground would be a non-Hispanic Black/African-American male who is a heavy smoker.

We asked participants to respond yes or no to the statement, “Cigarette butts are biodegradable”. Replies were equally distributed across “yes”, “no”, and “don’t know”. Women were much more likely (40%) than men (30%) to correctly believe butts are not biodegradable (*X*^2^ (1, *n* = 6045) = 433, *p* < 0.001), however, more women (38%) were unsure than men (25%). Men were more certain, but also more incorrect. There were differences among the racial groups (*X*^2^ (10, *n* = 7358) = 1164, *p* < 0.001). Whites and Black/African-American participants were equally likely to believe incorrectly that butts are biodegradable, but a larger percentage of White participants (36%) than Black/African-American participants (32%) knew butts were not biodegradable. Participants who knew that butts were not biodegradable were more likely to be bothered by seeing cigarette butts on the ground (*X*^2^ (2, *n* = 7532) = 351, *p* < 0.001). People who were unsure about biodegradability were more likely to disagree with the statement that cigarette butts are litter (*X*^2^ (1, *n* = 1866) = 64.9, *p* < 0.001).

*Relationship between attitudes and behavior.* Attitudes and behavior were related. People who were bothered by cigarette-butt litter were much more likely to have disposed of the last butt they smoked properly (*X*^2^ (6, *n* = 7532) = 568, *p* < 0.001). They disposed of fewer butts improperly in the past 24 h than those participants who were not bothered by cigarette butts on the ground (*X*^2^ (60, *n* = 7532) = 927, *p* < 0.001). Participants bothered by cigarette butts on the ground were less likely to have tossed a butt to the ground in the past month (*X*^2^ (6, *n* = 7532) = 1206, *p* < 0.001).

People who believed (correctly) that butts are not biodegradable were less likely to have ever tossed a butt out of a car window (*X*^2^ (2, *n* = 7532) = 96.8, *p* < 0.001) or toss a butt to the ground (*X*^2^ (2, *n* = 7532) = 96.3, *p* < 0.001). Those who believe butts are not biodegradable were also more likely to have not littered a butt in the past month (*X*^2^ (2, *n* = 7532) = 48.1, *p* < 0.001). They were also more likely to have disposed of the last cigarette butt they smoked outside properly (*X*^2^ (1, *n* = 7532) = 102, *p* < 0.001).

More women (74%) than men (69%) believed that cigarette butts are harmful to the environment (*X*^2^ (1, *n* =6409) = 65.6, *p* < 0.001). Hispanic participants (74%) were slightly more likely than non-Hispanic participants (71%) to believe this, but the result was not statistically significant (*X*^2^ (1, *n* =7292) = 3.6, *p* = 0.17). The strongest support came from Native Hawaiian and Pacific Islander participants (80%). They were the racial group most likely to believe that butts are harmful to the environment. Black/African Americans (69%) were the least likely to believe this. Black/African American participants were also less likely than White participants to support the statement that cigarette butts are harmful to the environment (*X*^2^ (1, *n* =7292) = 11.6, *p* = 0.003). People who reported smoking more were less likely to believe butts are harmful to the environment (*X*^2^ (1, *n* =6468) = 15.2, *p* = 0.004). Those who said they never tossed a butt on the ground were more likely to believe that butts are harmful to the environment (*X*^2^ (1, *n* =6468) = 80.4, *p* < 0.001). People who carry a personal pocket ashtray were more likely to believe that butts are harmful to the environment (*X*^2^ (3, *n* =6468) = 21, *p* < 0.001).

### 4.4. Logistical Regression Results

Following Rath et al. [29], we computed odds ratios from logistic regressions on two dependent variables: did you ever dispose of a cigarette butt on the ground or out a car window (Table 3), and did you litter a cigarette butt in the past month (Table 4). We also repeated the analysis for two other dependent variables: how did you dispose of the last cigarette butt you smoked outdoors (Table 5), and how many butts did you litter in the past 24 h (Table 6). For Table 3, Table 4 and Table 5, the multivariate model includes only those significant variables in bivariate analysis. Because the dependent variable in Table 6 was continuous, we used multiple linear regression on all variables. We combined the racial categories Asian and Hawaiian or Pacific Islander (PI) to make our results directly comparable to Rath et al.

For the first dependent variable, five of the independent variables were significantly related to ever littering in the multivariate analyses. Having an attitude that cigarette butts were bothersome was the most strongly related variable. Considering butts to be litter had the second-strongest relationship.

For past-month littering behavior, eleven variables were significant in the multivariate model. Again, the strongest effect was associated with the attitude of seeing cigarette butts as bothersome, followed by the belief that cigarette butts are litter (see Table 4).

Eight variables showed a significant relationship to how the respondent disposed of the last cigarette smoked outdoors (see Table 5). The effects were smaller than in the previous two models, but again, being bothered by seeing cigarette butts had the strongest relationship, followed by considering butts to be litter.

Table 6 shows the results of a multiple linear regression on the variable: How many butts did you litter in the past 24 h?

## 5. Discussion

### 5.1. Littering Rates and Gender

Our respondents reported that they littered 47% of the cigarette butts that they smoked. This is similar to numbers reported in other studies. Basto-Abreu et al. asked smokers in urban areas of Baja California, Mexico how many out of every ten cigarettes they toss to the ground [34]. Self-reports were that they littered 45% of cigarette butts they smoked. An observational study by Sibley et al. of 181 smokers on a college campus in New Zealand reported that 98.7% of cigarettes smoked were littered [27]. The largest observational study of smokers’ littering behavior in public found that 57% of people observed smoking littered one or more butts, and overall, 65% of cigarette butts were littered [26]. Smith and Novotny, in their summary of industry research reported smoker littering rates of 45–92% [35]. The differences among these studies may be explained by any number of factors including age, gender, cultural acceptance of littering behavior, availability of litter-collection containers, and weather. The differences among observational and self-report data suggest the possibility that people underestimate their littering behaviors. Overall, studies consistently show that smokers litter a large percentage of the cigarette butts they smoke.

While Schultz et al. did not find gender to be a significant predictor of littering cigarette butts, Rath et al. did [26,29]. Rath et al. found men more likely to have littered in the past month than women (OR = 1.49, 95% CI = 1.14–1.94). This was similar to our findings for the same question (OR = 1.30, 95% CI = 1.17–1.45). When we asked about littering cigarette butts ever, neither Rath et al. nor our study found a significant relationship with gender. We found stronger gender influences when we asked about how participants disposed of their last cigarette smoked outdoors (OR = 1.35, 95% CI = 1.22–1.50) and the number of cigarette butts tossed to the ground in the past 24 h (OR = 2.31, 95% CI = 2.03–2.61). 

### 5.2. Littering Rates and Demographics

Rath et al. is the only other study we know of that measured race and littering rates for cigarette butts [29]. They found significant associations for Black/African-American participants littering more than White participants, but only in the bivariate models. The effect disappeared in the multivariate model. We found the same results. For past-month littering, we found significantly lower rates for Asian/PI participants (OR = 0.60, 95% CI = 0.46–0.77) and those who declined to answer (OR = 0.62, 95% CI = 0.44–0.88). Rath et al., found no significant associations among race and littering in their multivariate model [29]. In general, the effect of race or ethnicity on cigarette-butt-littering behavior seems to be small.

### 5.3. Personal Pocket Ashtrays

We could find no other studies of smokers that reported on carrying a personal pocket ashtray. Presumably, people who carry one would be less likely to litter, simply because they have a readily available means to extinguish and carry the cigarette butt. We did find that people who carried a personal pocket ashtray were more likely to report lower littering rates. Interestingly, while Black/African-American participants were more likely than White participants to litter, they were also more likely to carry a personal pocket ashtray.

### 5.4. Beliefs about Cigarette-Butt Biodegradability

Presumably, a smoker who believes butts are biodegradable would be more likely to litter because they see the litter as temporary and not harmful. In their review of cigarette industry documents, Smith and Novotny reported on industry-funded studies that found that smokers widely believe that butts are not biodegradable [35]. Only one other non-industry study examined smokers’ beliefs about the biodegradability of cigarette butts and their littering behaviors [29]. They reported that 79% of smokers believe that butts are not biodegradable. We found much lower rates. Only 40% of women and 30% of men in our study believed that butts were not biodegradable. Rath et al., also found that people who believed (incorrectly) that butts are biodegradable were more likely to have ever littered (OR = 1.47, 95% CI = 1.02–2.13) [29]. Both our study (OR = 1.39, 95% CI = 1.25–1.55) and the Rath et al. study (OR = 1.58, 95% CI = 1.16–2.17) found that people who believe that butts are biodegradable are more likely to have littered in the past month.

### 5.5. Harmfulness of Butts to the Environment

Rath et al. measured respondents’ agreement with the statement: “Cigarette butts are harmless when eaten by animals/marine life”. They reported that 87% of smokers disagreed with the statement. We did not ask that exact same question but did find that 74% of women and 69% of men believe that cigarette butts are harmful to the environment. Clearly, most smokers believe that cigarette butts are harmful to the environment. There is considerable research documenting the negative effects of used cigarette butts in both terrestrial and aqueous environments. Future research could explore in more detail if smokers understand *why* used cigarette butts are harmful to the environment.

### 5.6. Attitudes and Behavior

We could find no other studies that measured smokers’ attitudes toward cigarette-butt litter. We asked respondents if they were bothered by cigarette-butt litter. This yielded the strongest association with littering in all four of our models. Given that attitudes are known to be precursors of intention to act, it is surprising that attitudes have not been measured in the past. One of the key findings of this study is that attitudes were the best predictor of reported behavior.

One of the most significant findings of the study by Rath et al. was that smokers who thought of cigarette butts as litter were 3.83 times (95% CI = 2.13–6.93) less likely to have ever littered and 3.68 times (95% CI 2.04 to 6.66) less likely to have littered in the past month. Of all the variables studied by Rath et al., this had the strongest influence on behavior. Whether we asked about ever littering, littering in the past month, littering the last cigarette butt, or rates of littering, we also found that people who have the attitude that butts are litter reported littering less, although the effect was not as strong as that reported by Rath et al. [29].

### 5.7. Limitations of This Study

Data reported here are based on a large, national sample, yet there are important methodological limitations to acknowledge. First, the sample is not necessarily representative of all college-aged smokers in the United States. The participant pool is self-selective in the sense that they volunteered to serve on a survey panel that was enlisted by Qualtrics. Second, self-report data are susceptible to several possible biases, including the social expectancy effect, wherein the participants’ reports are distorted to better represent what they believe is the proper or expected behavior.

### 5.8. Recommendations to Reduce Littering

There are several possible ways to reduce cigarette-butt littering. Two studies reported success by installing cigarette-butt-collection devices [36,37]. Morgan et al. found little success by adhering anti-littering messages to cigarette packs [30].

Models of environmental behavior seek to understand precursors and drivers of behavior so that unwanted behaviors can be mitigated. One of the best-known models is the theory of planned behavior (TPB) [38,39]. It suggests that the intention to perform a given behavior is a product of attitudes toward the behavior, associated normative beliefs, and perceived behavioral control. According to Ajzen, efforts to intervene in behavior should be directed at changing one of these three precursors. Another important model, value-belief-norm theory, posits that values and beliefs shape normative beliefs which then influence intention to act [40,41]. While VBN does not explicitly measure attitudes, it considers there to be a close relationship among values, beliefs, and attitudes [42,43]. Consequently, efforts to change behavior should focus on changing attitudes, values, beliefs, and norms.

Since negative attitudes about cigarette-butt litter and beliefs that butts are litter were the strongest predictors of behavior in this study, programmatic efforts to mitigate littering and marine debris ought to consider focusing on fostering this attitude and belief among smokers. Attitudinal change is the goal of much advertising [44]. One particularly interesting pathway to explore might be the use of humor to capture the target audience’s attention. Humorous messages are popular with advertisers because they are memorable and persuasive [45,46]. Humor affords the message-provider a valuable tool with cognitive or emotional appeal. By briefly holding an individual’s attention in a likeable, non-threatening way, humor enables a persuasive message to be received. Whether messages delivered in this manner can produce lasting changes in smokers’ attitudes or beliefs is a question for future research.

## 6. Conclusions

Despite the increased popularity of vaping, cigarette smoking is still a widely practiced behavior in college-aged Americans. Several studies have validated that smokers litter at least half of the cigarette butts that they smoke outdoors. These butts contain numerous toxic compounds, and when they are mobilized into waterways, they release these toxins into the water [13,47]. The cellulose acetate comprising cigarette filters breaks into smaller pieces but does not biodegrade under most conditions. Instead, it becomes available to marine life and finds its way into the food web. This is important because cellulose acetate can adsorb other toxic chemicals and transport these into marine life, where they can bioaccumulate and potentially affect humans [20,33].

This study asked 7532 college-aged, American smokers to self-report on their smoking and littering behaviors as well as their attitudes and beliefs about littering cigarette butts. A large majority of the smokers surveyed here litter cigarette butts regularly. The study found that negative attitudes toward littered cigarette butts was the strongest factor predicting littering of cigarette butts. The second-strongest factor was the belief that cigarette butts are litter.

Based on these results, it can be concluded that, to reduce cigarette-butt litter, interventions should focus on associating butts with litter and changing smokers’ attitudes toward cigarette-butt litter so that more people perceive them as bothersome.

## Figures and Tables

**Table 1:** Racial and ethnic makeup of sample (*n* = 7532).

Race ^1^	Hispanic	Non-Hispanic	Total	2020 Census 18+
American Indian or Alaska Native	3.4%	1.4%	1.8%	1.1%
Asian	2.5%	3.8%	3.6%	6.1%
Black or African American	15.7%	18.8%	18.2%	12.0%
Native Hawaiian or Pacific Islander	0.9%	0.3%	0.4%	0.2%
Mixed	18.3%	2.7%	6.1%	16.5%
White	52.4%	72.6%	67.6%	64.1%
I decline to answer	6.7%	0.5%	2.3%	0%
Grand Total	20.4%	76.5%	100.0%	100.0%

^1^ https://www.census.gov/library/stories/2021/08/improved-race-ethnicity-measures-reveal-united-states-population-much-more-multiracial.html, accessed on 25 January 2022.

**Table 2:** Number of cigarettes smoked by participants per day (*n* = 7532).

Cigarettes Smoked Per Day	Percent
1–5	44.4%
6–10	31.8%
11–15	14.0%
16–20	5.9%
20+	4.0%

**Table 3:** Statistically significant predictors of ever littering ^†^ cigarette butts among college-age smokers (*n* = 7532, *p* < 0.05).

	Bivariate		Multivariate	
	OR	95% CI	OR	95% CI
**Average number of cigarettes smoked a day**	1.40	(1.32–1.49)	1.38	(1.30–1.47)
**Do you carry a pocket ashtray?**				
Always	0.62	(0.54–0.71)	0.81	(0.68–0.95)
Most days	0.80	(0.71–0.90)		
Never	Ref		Ref	
**Seeing cigarette butts on the ground bothers me**				
Strongly disagree/Disagree/Somewhat disagree/DK	5.91	(5.17–6.75)	5.11	(4.43–5.89)
Strongly agree/Agree/Somewhat agree	Ref		Ref	
**Cigarette butts are biodegradable**				
No/DK	Ref			
Yes	1.28	(1.14–1.43)		
**Do you consider cigarette butts to be litter?**				
No/DK	2.58	(2.18–3.05)	1.73	(1.44–2.07)
Yes	Ref		Ref	
**Cigarette butts are harmful to the environment**				
No/DK	Ref		1.20	(1.03–1.40)
Yes	2.16	(1.88–2.48)	Ref	
**Gender**				
Male	1.12	(1.00–1.25)		
Female	Ref			
**Race**				
White	Ref			
Black AA	1.24	(1.07–1.44)		
Asian or PI	0.65	(0.51–0.84)		
I decline to answer	0.60	(0.44–0.83)		

^†^ Ever littering = Have you ever disposed of a cigarette butt on the ground or out of a car window? OR = Odds ratio; Ref = referent.

**Table 4:** Statistically significant predictors of past-month cigarette-butt littering ^†^ among college-age smokers (*n* = 7532, *p* < 0.05).

	Bivariate		Multivariate	
	OR	95% CI	OR	95% CI
**Average number of cigarettes smoked a day**	1.23	(1.18–1.29)	1.23	(1.17–1.29)
**Do you carry a pocket ashtray?**				
Always	0.42	(0.37–0.47)	0.40	(0.34–0.47)
Most days	0.66	(0.59–0.73)	0.53	(0.46–0.61)
Occasionally	1.29	(1.15–1.44)	0.79	(0.68–0.91)
Never	Ref		Ref	
**Seeing cigarette butts on the ground bothers me**				
Strongly disagree/Disagree/Somewhat disagree/DK	4.62	(4.16–5.14)	3.66	(3.26–4.10)
Strongly agree/Agree/Somewhat agree	Ref		Ref	
**Cigarette butts are biodegradable**				
No/DK	Ref		Ref	
Yes	1.44	(1.31–1.59)	1.39	(1.25–1.55)
**Do you consider cigarette butts to be litter?**				
No/DK	1.69	(1.49–1.92)	1.15	(1.00–1.33)
Yes	Ref		Ref	
**Cigarette butts are harmful to the environment**				
No/DK	Ref		1.23	(1.08–1.41)
Yes	2.00	(1.78–2.24)	Ref	
**Gender**				
Male	1.11	(1.01–1.23)	1.30	(1.17–1.45)
Female	Ref		Ref	
**Race**				
White	Ref		Ref	
Asian or PI	0.57	(0.45–0.71)	0.60	(0.46–0.77)
I decline to answer	0.66	(0.49–0.90)	0.62	(0.44–0.88)
**Ethnicity**				
Non-Hispanic/I decline to answer	Ref		Ref	
Hispanic	1.21	(1.08–1.36)		

OR = Odds ratio; Ref = referent. ^†^ Past-month littering = Disposing of cigarette butts on the ground, in a sewer/gutter, or down a drain within the last 30 days.

**Table 5:** Statistically significant predictors of littering ^†^ the last cigarette smoked among college-age smokers (*n* = 7532, *p* < 0.05).

	Bivariate		Multivariate	
	OR	95% CI	OR	95% CI
**Average number of cigarettes smoked a day**	1.20	(1.15–1.25)	1.17	(1.12–1.22)
**Do you carry a pocket ashtray?**				
Most days	0.85	(0.76–0.95)		
Never	Ref		Ref	
**Seeing cigarette butts on the ground bothers me**				
Strongly disagree/Disagree/Somewhat disagree/DK	2.52	(2.28–2.77)	2.25	(2.03–2.50)
Strongly agree/Agree/Somewhat agree	Ref		Ref	
**Cigarette butts are biodegradable**				
No/DK	Ref		Ref	
Yes	1.56	(1.41–1.73)	1.39	(1.25–1.55)
**Do you consider cigarette butts to be litter?**				
No/DK	1.92	(1.71–2.15)	1.42	(1.25–1.60)
Yes	Ref		Ref	
**Cigarette butts are harmful to the environment**				
No/DK	Ref		1.25	(1.12–1.41)
Yes	1.84	(1.66–2.04)	Ref	
**Gender**				
Male	1.33	(1.21–1.46)	1.35	(1.22–1.50)
Female	Ref		Ref	
**Race**				
White	Ref		Ref	
Black AA	1.29	(1.14–1.45)		
Asian or PI	0.59	(0.45–0.77)	0.67	(0.51–0.88)
I decline to answer	0.62	(0.44–0.87)	0.67	(0.47–0.95)

OR = Odds ratio; Ref = referent. ^†^ Littering = Disposing last cigarette butt smoked outside on the ground, out of a car window, or in a sewer.

**Table 6:** Statistically significant predictors of number of cigarette butts littered ^†^ in past 24 h among college-age smokers (*n* = 7532, *p* < 0.05).

	Multiple Linear Regression	
	OR	95% CI
**Average number of cigarettes smoked a day**	2.35	(2.23–2.49)
**Do you carry a pocket ashtray?**		
Always	3.48	(2.89–4.20)
Most days	2.40	(2.05–2.81)
Occasionally	1.40	(1.21–1.64)
Never	Ref	
**Seeing cigarette butts on the ground bothers me**		
Strongly disagree/Disagree/Somewhat disagree/DK	2.59	(2.28–2.94)
Strongly agree/Agree/Somewhat agree	Ref	
**Cigarette butts are biodegradable**		
No/DK	Ref	
Yes	1.47	(1.30–1.66)
**Do you consider cigarette butts to be litter?**		
No/DK	2.14	(1.84–2.49)
Yes	Ref	
**Cigarette butts are harmful to the environment**		
No/DK	Ref	
Yes	1.30	(1.13–1.49)
**Gender**		
Male	1.79	(1.59–2.02)
Female	Ref	
**Race**		
White	Ref	
Black AA	1.30	(1.11–1.51)
Asian PI		
Mixed	0.65	(0.51–0.84)
I decline to answer	0.59	(0.40–0.87)

OR = Odds ratio; Ref = referent. ^†^ Number of cigarette butts discarded on the ground, out of a car window, or in a sewer in the past 24 h.

## Data Availability

Data are being archived at ICPSR at the University of Michigan https://www.icpsr.umich.edu, accessed on 24 June 2022.
